# Oral Health Literacy and Persistent Oral Health Inequalities in Older Adults: A Conceptual Model

**DOI:** 10.3390/geriatrics11050146

**Published:** 2026-10-15

**Authors:** Hendrik-Matthias Pallien, Madhat Alrayes, Adrian Niemuth, Stefan Wahl, Julien Lochmann, Fabian Renger, Attila Czirfusz, Viktor Foltin

**Affiliations:** Department of Public Health, St. Elizabeth University of Health and Social Work, 810 06 Bratislava, Slovakia

**Keywords:** oral health, aged, public health, health literacy, healthcare disparities, health services to the aged

## Abstract

**Background:** Oral health inequalities in older adults persist despite the availability of effective preventive and clinical interventions, representing a significant public health challenge in ageing societies. **Objective:** This narrative review examines how oral health literacy (OHL) and structural barriers interact to sustain these inequalities and proposes a conceptual framework to guide future research and intervention design. **Methods:** A narrative synthesis of peer-reviewed literature and authoritative policy documents published between January 2015 and October 2025 was conducted across PubMed, Scopus, Web of Science and Google Scholar. Older foundational studies were included where conceptually relevant. Findings were organised thematically into three categories: oral health literacy, structural barriers and their interaction within a social–ecological framework. **Results:** Low OHL constrains self-care capacity, symptom recognition and navigation of dental and medical systems. Structural barriers including inadequate insurance coverage, workforce shortages, geographic inaccessibility and fragmented care models restrict access independently of individual knowledge or motivation. The two factors interact in a mutually reinforcing manner: low OHL amplifies the impact of structural constraints, while structural exclusion suppresses OHL development. This dynamic sustains a cycle of unmet need, preventable disease, systemic health complications and reduced quality of life. Promising intervention strategies include integration of oral health into primary and long-term care, teledentistry, targeted literacy programmes, and policy reform expanding dental insurance coverage. **Conclusions:** Reversing oral health inequalities in older adults requires a shift from reactive, treatment-centred models toward proactive, integrated, and equity-oriented approaches that address individual capacity and structural conditions simultaneously.

## 1. Introduction

The world’s population is ageing at an unprecedented rate, and this places a growing strain on healthcare systems everywhere [1]. As people live longer, maintaining health and quality of life matters more than ever, yet oral health remains one of the most persistently neglected aspects of overall well-being [2]. Contrary to a common assumption, growing old does not inevitably bring tooth loss or oral disease; outcomes depend heavily on modifiable risk factors and, above all, on access to appropriate care [3]. Several conditions cluster in later life. Dental caries, periodontal disease, xerostomia and oral cancer are especially common among older adults and bring severe pain, impaired function and a heavy financial burden [4]. The epidemiology bears this out: moderate to severe periodontitis is highly prevalent in older populations worldwide [5], and German DMS V data show that roughly half of community-dwelling adults aged 65 to 74 are affected [6]. Xerostomia, for its part, troubles around 30% of older adults taking multiple medications [5]. Access to preventive care is constrained as well, since sizeable proportions of older adults in many countries lack adequate dental insurance [7]. The consequences reach well beyond the mouth. Poor oral health has been linked to a range of systemic conditions, among them cardiovascular disease, impaired glycaemic control in diabetes, aspiration pneumonia and malnutrition [8,9,10]. It also shapes social life and mental health: difficulty eating, speaking or interacting with others can foster isolation, depression and a diminished quality of life [7]. Despite the scale of all this, oral healthcare for older adults continues to suffer from systemic neglect and inequity. Existing care models are often poorly suited to older patients, particularly those with multimorbidity, functional disability or a place in residential care [2,11]. The gap has hardened into a compounding public health crisis, and healthcare systems now face an urgent need to rethink how oral care is delivered to ageing populations, with far closer attention to equity, coordination and sustainability. This review draws together the existing evidence on that challenge, examines how oral health literacy and structural barriers interact, weighs the implications for population health and considers the intervention strategies that might help. The central question addressed is: how do low oral health literacy and structural barriers interact to sustain oral health inequalities in older adults and what conceptual framework can capture this interaction to guide future research and intervention?

## 2. Materials and Methods

This narrative review brings together current evidence on oral health inequalities in older adults, with particular attention to oral health literacy (OHL) and structural barriers. A narrative format was chosen deliberately, because the research question is conceptual and integrative and draws on heterogeneous evidence from across dentistry, gerontology, public health and health policy. A structured search of PubMed, Scopus, Web of Science and Google Scholar was carried out between June and October 2025. Search terms combined phrases such as “oral health literacy,” “older adults,” “elderly oral health,” “dental care access,” “structural barriers,” “oral health inequalities,” “geriatric dentistry,” “long-term care” and “teledentistry.” Boolean operators (AND, OR) and standard truncation were applied to improve sensitivity, and the reference lists of key papers and relevant systematic reviews were screened by hand to capture further sources.

Studies were eligible if they were (i) peer-reviewed articles, systematic reviews or authoritative policy documents published in English; (ii) published between January 2015 and October 2025; and (iii) focused on adults aged 60 years or older, or on population-level data reported separately for this group. Older foundational works from before 2015 were kept where they were conceptually essential, especially for theoretical models such as the social determinants of health framework. Studies were excluded if they (i) dealt exclusively with paediatric or younger adult populations; (ii) were case reports or commentaries lacking an empirical or systematic basis; or (iii) were non-English publications without an authoritative translation.

Studies were considered for inclusion if they addressed:

- Oral health outcomes of older adults.
- The factors that influence oral health literacy.
- Structural, financial or systemic factors that prevent access to dental care.
- Interventions to tackle inequalities in oral health in older populations.

In keeping with narrative review methodology, no formal critical appraisal tool was applied to individual studies, though the internal quality assessments of any cited systematic reviews were taken into account. A critical interpretive approach guided the identification of themes, patterns and conceptual relationships across the literature, and the findings were then organised thematically into three categories: oral health literacy, structural barriers and their interaction within a social–ecological framework. Theme development was inductive and interpretive rather than based on a formal qualitative coding protocol: sources were read in full, recurring constructs were grouped by the authors into candidate categories, and these were iteratively consolidated into the three themes reported here; no software-assisted or independently double-coded content analysis was undertaken. Because it draws substantially on existing systematic and scoping reviews alongside primary studies, the review offers broad coverage of a mature and rapidly growing body of literature. In keeping with narrative review methodology, and consistent with the SANRA guidance for narrative reviews, the identification and inclusion of sources were guided by the authors’ subject-matter expertise rather than a formal, reproducible search-and-screening protocol. Accordingly, no PRISMA-style selection flowchart or database-specific search syntax is reported, and a degree of selection subjectivity is acknowledged as an inherent feature of the narrative approach. The principal sources drawn on this review, with their study design, country or region and care setting, are summarised in Table 1.

## 3. Conceptual Framework

Making sense of oral health in later life calls for a conceptual framework capable of holding the full complexity of the problem. The framework set out in this section serves as the analytical lens through which the thematic findings in Section 4 are interpreted; presenting it first makes explicit the theoretical assumptions that organise the synthesis. This review takes a social–ecological perspective, treating health outcomes as the product of dynamic interactions across several levels: individual knowledge and skills, interpersonal and organisational practices, community resources and broader policy. Such a view extends the biomedical model by acknowledging how social determinants and system structures shape both health status and access to care [32]. The first core concept underpinning the model is this holistic, social–ecological understanding of oral health in later life. A few points follow from it. First, oral health in older age is best understood holistically, as something more than the absence of caries or periodontal disease. For an older person, it is what makes everyday life possible: eating, speaking, socialising and smiling without pain, discomfort or embarrassment [4,33]. It includes the natural teeth, the periodontal tissues, the oral mucosa and the jaw bones, along with any prosthetic replacements such as dentures or implants. Second, the oral health risks of older adults differ from those of younger people [12]. A distinctive risk profile emerges from age-related physiological change such as reduced salivary flow, xerostomia brought on by polypharmacy, the cumulative toll of past disease and treatment and comorbidities like dementia, stroke or arthritis that erode the capacity for self-care [10]. These two features (an expanded, function-centred definition of oral health and an age-specific risk profile) are not incidental background: they delimit what the model seeks to explain and why older adults constitute a distinct case, and they are carried forward into the two core determinants set out below.

A second core concept is oral health literacy (OHL). Building on the wider notion of health literacy, OHL describes how individuals can obtain, process and understand basic oral health information and services well enough to make sound health decisions [34]. In older adults, it is multidimensional. It spans an awareness of ageing-related oral health risks and of one’s own medical history; the ability to carry out effective daily oral care, often with adaptive aids that make up for reduced dexterity; the capacity to navigate tangled health and insurance systems in order to reach and afford care; and clear communication with dental and medical professionals [13]. OHL is therefore far more than reading comprehension. It is a crucial link between health knowledge, the uptake of preventive behaviours and successful engagement with the care system. Individual OHL must, however, be distinguished from organisational health literacy, that is, the degree to which health and dental services are designed to be easy to reach, understand and navigate. Technically complex materials, professional jargon and fragmented systems are properties of services rather than deficits of the individual and therefore belong conceptually among the structural barriers described below. The third core concept is that of structural barriers.

These are the systemic, often institutionalised determinants that limit access to and use of oral healthcare, and they work largely independently of any individual’s knowledge, motivation or perceived need [2,35]. Embedded in the economic, political and organisational fabric of healthcare systems, they take several forms. The most significant is financial: in most countries, routine dental care sits outside public health insurance for older adults, leaving them to meet high out-of-pocket costs on a shrinking retirement income [36]. Workforce and geographic barriers add to the picture [15]. There are documented shortages of dental professionals trained in geriatric dentistry, together with a marked maldistribution of services that leaves rural and underserved urban areas as dental deserts [15]. Service delivery falters too, through physically inaccessible facilities, weak integration between oral health and primary medical care and the absence of robust, enforceable oral care standards in settings such as nursing homes [18]. Policy and attitudinal barriers deepen the problem: pervasive ageism helps push oral health down the list of priorities for older adults, while the long-standing separation of oral health from general health in policy, professional training and reimbursement frameworks has entrenched that neglect [37]. The theoretical grounding of this review lies in the social determinants of health tradition, which holds that health inequalities persist because individual-level capacities and the structural conditions built into healthcare systems interact in systematic ways [38]. Seen through this lens, oral disease in later life is not an isolated clinical event but the endpoint of a life-course accumulation of exposures, shaped by socioeconomic status, educational attainment, ethnicity and geographic location, all of which govern both exposure to risk and access to care [38]. On this foundation, and drawing on evidence that dental service use among older adults owes more to systemic and organisational factors than to individual need alone [16], the review advances a specific proposition: that oral health literacy (OHL) and structural barriers are not two independent, additive determinants of oral health but mutually reinforcing forces whose combined effect exceeds the sum of their parts.

The model’s distinctive contribution lies in spelling out how that coupling actually works. Low OHL narrows an older adult’s ability to navigate an already fragmented system, to make sense of eligibility and cost information and to advocate for care; structural barriers, for their part, strip away the very opportunities through which literacy might be exercised or built, so that each deficit amplifies the other rather than simply adding to it [14,16]. This reciprocal dynamic should be sharpest among frail and institutionalised older adults, for whom reduced physiological and functional reserves lowers the threshold at which an unmet need tips into rapid oral health deterioration [24], and for whom the burden of untreated disease is empirically concentrated [19,31]. Oral health outcomes in later life are therefore best read as products of this interaction rather than of either factor taken alone. The novelty of the model lies less in naming either determinant, since both are well described individually, than in specifying their coupling: it treats OHL and structural barriers as a single, non-additive system, links that synergy explicitly to functional-status stratification (Table 2) and to rapid oral health deterioration, and thereby yields testable propositions rather than a static inventory of risk factors. A schematic representation of the model, covering the upstream social determinants, the interaction between OHL and structural barriers, the resulting health and social outcomes and corresponding intervention domains, is provided in Figure 1.

## 4. Results: Thematic Findings

The synthesis is presented below under three thematic headings: oral health literacy, structural barriers, and their interaction within a social–ecological framework.

### 4.1. Oral Health Literacy in Older Adults

Effective self-care rests on adequate oral health literacy, yet OHL tends to be low among older adults. Several things contribute, among them generational gaps in formal education, cognitive impairment and age-related sensory decline [13].

Higher OHL goes hand in hand with better oral health behaviours, such as regular toothbrushing and routine dental visits, and with better clinical outcomes, including greater tooth retention and oral health-related quality of life (OHRQoL) [13,28]. In a recent cross-sectional study of community-dwelling older adults, roughly 44% showed low oral health literacy, and higher OHL predicted fewer missing teeth and better oral health-related quality of life [39]. Low OHL pulls in the opposite direction. It is bound up with misconceptions, such as the belief that losing teeth is a normal part of ageing or that dentures do away with any further need for dental care, and both ideas dampen the uptake of preventive services [13]. Cognitive impairment, dementia or Alzheimer’s disease above all, do much to erode functional OHL, undermining the ability to keep up hygiene routines, recognise symptoms and follow care instructions [20]. The provider side of literacy matters just as much: medical jargon and dense written materials often get in the way of communication, raising barriers to comprehension and adherence for older patients [21]. These provider- and system-side obstacles reflect limited organisational health literacy and are best understood as structural rather than individual limitations.

These effects can be measured. Studies show that OHL independently predicts both self-reported dissatisfaction with oral health and the use of dental services, with low OHL often accompanying care that is put off until problems grow severe and expensive [14,16]. That delay directly weakens an older person’s ability to surmount the structural barriers built into healthcare systems.

### 4.2. Structural Barriers

Individual factors like OHL matter, but they play out within an environment defined by structural barriers, and it is these that impose the tightest constraints on oral health equity in older populations. Such system-level failures block access to care regardless of an older adult’s knowledge, perceived need or motivation to seek help. The barrier reported most often is financial [17]. In many high-income countries, public insurance schemes for older adults simply leave routine dental care out. The United States Medicare programme, for instance, excludes routine dental check-ups, preventive care, dentures and most oral surgery. Comparable gaps appear across several European and Asian systems, albeit in different configurations, and the upshot is that older adults must often buy costly private cover, pay out of pocket or go without. Systematic reviews return to the same finding: a lack of dental insurance is among the strongest predictors of low utilisation and unmet dental need in older populations [7,17]. Workforce and geographic constraints sit close beside these financial ones. The international shortage of dentists trained in geriatric dentistry is well documented, and few practitioners are equipped to treat frail or institutionalised older patients [15]. Those who are available tend to be unevenly spread, so that rural areas and low-income urban districts often become dental deserts. Even when a dentist is within reach, the building itself can be the obstacle: many practices lack wheelchair ramps, accessible entrances, treatment rooms that can take mobility aids and chairs suited to patients who cannot move easily [17]. And for an older adult who no longer drives and has no reliable, affordable public transport, getting to an appointment at all may be out of the question.

One of the most deep-rooted failures is structural: oral healthcare has long been kept apart from the rest of the health system. Medical and dental services run in parallel, with separate records, distinct funding streams, little professional contact and minimal coordination of care [40]. Primary care physicians and nurses, the very people who see older adults with chronic conditions most often, usually receive little or no training in basic oral health screening [40]. They may therefore miss the early signs of oral disease or fail to appreciate how it bears on systemic conditions such as diabetes, where periodontal status is an established modifier of glycaemic control [9]. Dentists, in turn, frequently work without a full medical history or an up-to-date medication list, which hampers informed treatment planning. The fragmentation squanders real opportunities for early detection, patient education and timely referral, and it undermines the safety and continuity of care.

Nowhere are these failures more visible than in long-term care (LTC) settings such as nursing homes and assisted living facilities. Residents there tend to have the greatest oral health needs and the least capacity for self-care, and yet oral health is routinely pushed aside [41]. The barriers stack up at several levels. Frontline staff often get little training in oral hygiene techniques or in why oral care matters systemically, and, stretched thin by competing demands, they come to treat oral hygiene as an optional extra [20]. The clinical cost of this is plain in the work of Mirwald and Foltin (2025): in a stratified comparison of community-dwelling, partially dependent and institutionalised older adults, periodontitis was far more prevalent in the institutionalised group [31]. Residents with cognitive impairment or dementia may resist oral care, and untrained staff often lack the skills to manage such situations safely [20]. Regulatory oversight of oral care in LTC stays weak, mirroring its low standing on institutional and policy agendas [41]. The cumulative result is a well-documented burden of poor oral health among institutionalised older adults: heavy plaque, untreated caries, periodontal disease and chronic oral pain [24]. Oral frailty, the functional decline of the mouth and dentition, is especially common in this group and carries with it reduced quality of life and worse health outcomes [22]. Patterns of dental service use shift as well once home care or institutional care begins, with utilisation falling even as clinical need climbs [42].

### 4.3. The Interaction Between Oral Health Literacy and Structural Barriers

The link between low OHL and structural barriers is best seen as an interaction rather than a matter of two separate influences. The two reinforce one another, and where they coincide, they tend to feed a cycle that speeds oral health decline and grows steadily harder to break. The literature does not yet allow this interaction to be quantified precisely, but the conceptual and clinical evidence points consistently to a non-additive relationship.

This mutually reinforcing interaction should be read as a conceptual proposition advanced by the model and still awaiting direct empirical test; by contrast, the component associations are each supported by primary and review-level evidence, namely the link between low OHL and poorer behaviours or dissatisfaction and the link between structural barriers and reduced utilisation. Picture an older adult with low OHL who assumes that tooth loss is inevitable and that dentures are the eventual answer. Someone in that position is unlikely to prize regular dental visits, and therefore unlikely to make the effort needed to get past financial or logistical obstacles. Early symptoms may go unrecognised as warning signs, and care is delayed further still. The same person may also lack the know-how to find subsidised community clinics, complete public assistance applications or press their case within a complex bureaucracy [13].

Clinically, older adults who are functionally dependent or institutionalised consistently fare worse than their independent peers, which fits the proposed model, since dependency magnifies both literacy deficits and structural barriers. The opposite case is just as telling. Take an older adult with high OHL who understands periodontal disease, spots the early signs of gingivitis and knows the worth of regular check-ups, but who lives on a modest pension in a rural area [27]. State insurance does not cover periodontal care, the nearest dentist is half an hour away and driving is no longer an option. Here strong literacy allows an accurate self-assessment but opens no practical route to care; cost, geography and transport cancel the literacy advantage out. This adverse synergy is not spread evenly. Systematic reviews report markedly worse oral health among institutionalised compared to community-dwelling older adults, a reflection of the way literacy deficits and structural access barriers pile up together [19]. The model proposed here helps make sense of that: institutionalised older adults meet the lowest baseline OHL and the most restrictive barriers at once, so both drivers concentrate in the same population. The dynamic bears down hardest on groups already at the margins [25]. Older adults from lower socioeconomic backgrounds, ethnic minorities and those in rural or remote areas contend with lower baseline OHL and heavier exposure to structural constraints such as poverty, limited insurance coverage and reduced service availability [25]. Jamieson and colleagues (2024) found that long-standing oral health disparities between racial and ethnic groups in Australia and the United States remain substantial and are still sustained by structural and socioeconomic factors [25]. A simple stratification of older adults by functional status helps show how literacy deficits and structural barriers accumulate (Table 2). It follows classifications used in the geriatric dentistry literature [19] and aligns with the empirical groupings reported by Mirwald and Foltin (2025) [31].

The barriers pile up most heavily in Group C, which goes some way toward explaining the disproportionately poor oral health seen there. Sharp, rapid declines in oral health can strike older adults caught between low OHL and entrenched structural barriers. Desai and Nair (2023) call this Rapid Oral Health Deterioration (ROHD) [24]. It is usually set off by an acute event, such as illness, a sudden jump in dependency, the side effects of new medication or the loss of a caregiver. In such moments, pre-existing low OHL and structural barriers do not cause ROHD directly; rather, they strip away the buffering capacity that would normally let an older adult absorb the shock, leaving them open to rapid deterioration over a short span. What follows is a self-reinforcing loop. Structural barriers restrict access to professional care, which cuts off tailored oral health education and early intervention, which in turn keeps OHL low and lets disease advance unchecked. Downstream come pain, infection, tooth loss, embarrassment, social withdrawal, falling self-efficacy and a reduced capacity to engage with health systems, and together these steadily wear away functional health literacy. Breaking the loop calls for interventions that work on the individual and structural dimensions at once [43].

## 5. Discussion

A central reason these disparities endure is that interventions rarely tackle individual and systemic determinants together. Literacy programmes on their own can raise knowledge but cannot dismantle financial or access barriers, while widening service availability without matching investment in OHL usually ends in underuse. That persistent gap between information, access and use keeps old inequalities alive. The knock-on effects of neglecting oral health in ageing populations are far-reaching, touching both health and social care systems. On the side of systemic health and expenditure, oral health is itself a risk factor for several serious conditions: periodontal disease is tied to cardiovascular complications and poorer glycaemic control, and aspiration of oral pathogens can bring on life-threatening pneumonia in frail older people [10]. Longitudinal data go further, marking poor oral health as an independent predictor of adverse geriatric outcomes including functional decline, sarcopenia, falls and hospitalisation [26]. Tooth loss and oral pain narrow what a person can eat, raising the risk of malnutrition and frailty [8], and treating dental emergencies in hospital is a costly, inefficient use of acute care [2]. Preventive care, by contrast, is reliably more cost-effective than emergency or restorative treatment. Oral health matters for psychosocial well-being too. Trouble with chewing, speaking and appearance is strongly bound up with social withdrawal, loss of dignity, depression and lower life satisfaction [27], an erosion of social connection that cuts against everything healthy ageing stands for. The damage, moreover, falls unevenly, weighing most on groups the healthcare system has already sidelined and thereby widening inequalities that principles of equity and social justice would have resolved [16,25]. Read through the social determinants of health framework [38], these patterns map onto a structural and intermediary pathway: socioeconomic position, education and geography act as structural determinants that shape both the development of OHL and exposure to organisational barriers, while OHL and self-care operate as intermediary determinants translating social position into concrete oral-health outcomes. Through this lens, the persistence of inequality is not a failure of individual behaviour but the predictable product of structural conditions acting through intermediary pathways, which implies that equity-oriented action must be proportionate to need rather than uniform.

Meeting this challenge takes multi-level strategies aimed at both individual capacity and the structural environment. Folding oral health into general and long-term care is a necessary first step towards breaking down the silos, and it means training primary care and LTC staff to carry out basic oral screening and to support daily oral hygiene [40,44]. Systematic reviews suggest that the most effective route in LTC is a multifaceted one, combining staff education, environmental adjustments and leadership support [23].

Teledentistry holds real promise for getting past barriers of geography and mobility, supporting remote consultations, triage, monitoring and patient education while sparing patients the journey [29]. Satellite-based models have already proved their worth in reaching remote communities, as work in rural Chile shows [30]. The digital divide remains a genuine constraint, though: older adults with limited digital literacy, patchy internet access or sensory impairments may struggle with these platforms unless they are given dedicated support, and any rollout should plan for that from the outset. On policy and financing, the single most powerful move is to widen public dental coverage for older adults. Sustained advocacy is needed to see comprehensive dental benefits written into national health programmes for older citizens, removing the chief financial barrier to care. On the workforce side, training in geriatric dentistry deserves active encouragement. OHL, too, can be built up through community-based efforts such as mobile clinics and health promotion tailored to older people, whose oral health-related quality of life stays stubbornly poor and in clear need of improvement [28]. Structured educational interventions have themselves been shown to improve oral health outcomes in older people [45]. Non-governmental organisations (NGOs) add a complementary layer, serving as providers, educators and advocates for disadvantaged older populations and bridging gaps that established systems so often leave open [46]. For all the growing literature on geriatric oral health and the rising policy attention, important evidence gaps still hold back the design, scaling and implementation of effective interventions. First, the field needs sturdier research on how well interventions work and what they cost. Pilot programmes and qualitative studies hint that integrated care, teledentistry and LTC staff training are promising, but high-quality longitudinal studies and randomised controlled trials remain thin on the ground, which limits what can be said about long-term outcomes, cost-effectiveness and return on investment [23]. Encouragingly, recent randomised trials of tailored oral-hygiene instruction and technology-assisted, augmented-reality oral-health education in older adults report short-term gains in hygiene, knowledge and self-efficacy [47,48]. Future studies should reach past intermediate measures like staff knowledge or plaque scores to hard endpoints: hospitalisation for pneumonia, glycaemic control in diabetes, nutritional status, total healthcare costs and patient-reported quality of life. Second, more implementation science is called for. Proving efficacy in a controlled trial is not enough; evidence is needed on how to scale successful pilots, embed them in existing health and social care systems, secure durable funding and train the wider healthcare and long-term care workforce [23]. Understanding the contextual factors that tip implementation towards success or failure matters every bit as much.

Third, research would benefit from a firmer equity orientation and from leaning more on disaggregated analysis. Much of the current evidence still treats older adults as a single undifferentiated group, and there is a pressing need to focus on underserved subgroups: older people with moderate-to-severe dementia, racial and ethnic minorities, the oldest older adults (over 85) and those experiencing homelessness [19]. Rather than merely cataloguing disparities, research should set about designing, testing and evaluating culturally competent, context-specific interventions for high-need groups. Finally, the evidence base is geographically lopsided. Most high-quality work has come out of high-income Western settings, and studies from low- and middle-income countries are urgently needed to develop and test affordable, culturally appropriate models of geriatric oral healthcare that can function where resources are tight. Closing these gaps is essential to building a more balanced, effective global response to the oral health needs of ageing populations.

This review offers a detailed synthesis of the available evidence, though several limitations warrant mention. First, the narrative method is inherently non-systematic. It allows an interdisciplinary overview of a complex topic, but it does not follow the structured search and selection procedures of a systematic review, which introduces a degree of selection bias, since the evidence base reflects the authors’ reading and the visibility of the literature rather than an exhaustive survey of everything published. Because no formal search-and-screening protocol or independent dual study selection was applied, the review is also open to publication and language bias; and because no quantitative synthesis or structured risk-of-bias appraisal was performed, the relative strength of individual associations cannot be weighted and the proposed model cannot be tested statistically within this review. Second, much of the evidence gathered here is observational, drawn from cross-sectional surveys, cohort studies and qualitative research; such work is valuable for identifying associations, patterns and lived experience, but it cannot establish causation. Third, the review leans mainly on literature from high-income Western countries, with most cited studies set in the United States, Europe, Australia or Thailand, which limits how far the findings carry to low- and middle-income settings, where health system structures, cultural contexts and available resources differ markedly. Finally, geriatrics, dentistry and health technology are all moving quickly. Recent as the cited sources are, the pace of change in policy, technology and practice means some emerging developments may not yet have reached the literature.

## 6. Conclusions

The burden of oral disease in older adults is no inevitable consequence of ageing. As this review has shown, it stems in large part from the reinforcing interplay between inadequate oral health literacy and deeply embedded structural barriers in healthcare financing, workforce and delivery. Neither factor alone accounts for why oral health inequalities persist; together they sustain a cycle of unmet need, preventable disease and avoidable systemic harm that falls disproportionately on the most vulnerable members of ageing populations. Breaking that cycle demands a decisive change of approach. Reactive, treatment-centred and professionally siloed models of care must give way to ones that are proactive, person-centred and integrated. That means coordinated action on several fronts at once: expanding oral health literacy through tailored, accessible programmes for older adults and their carers; reforming insurance policy so that essential dental services are universally covered; investing in the geriatric dentistry workforce and harnessing technologies such as teledentistry to widen reach; and building oral health systematically into primary care, chronic disease management and long-term care standards.

## Figures and Tables

**Figure 1:**
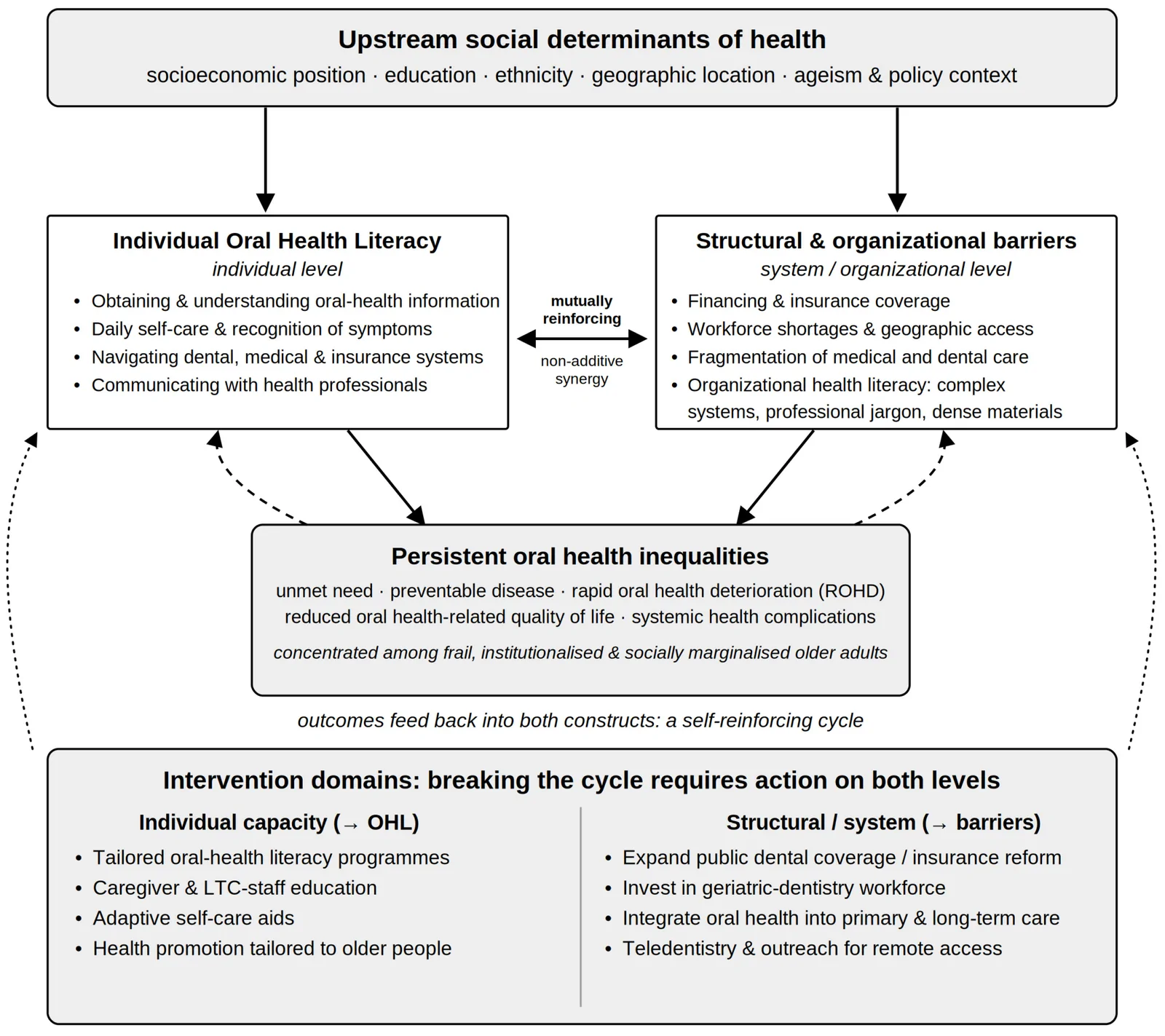
Conceptual model linking individual oral health literacy and structural/organizational barriers to persistent oral health inequalities in older adults. Upstream social determinants of health (**top**) shape two interacting levels: individual oral health literacy (**left**) and structural and organizational barriers (**right**). Arrow conventions: solid arrows denote directional influence (social determinants acting on each level, and both levels contributing to persistent oral health inequalities); the double-headed arrow between the two levels indicates their mutually reinforcing, non-additive interaction; dashed arrows represent feedback from persistent inequalities back to both levels, forming a self-reinforcing cycle; dotted arrows indicate the intervention domains (**bottom**) through which action on each level can break the cycle.

**Table 1:** Overview of the principal sources synthesised in this narrative review, by thematic focus. Study design and population were extracted from the source records; the table indicates the provenance of the review’s claims and is not a formal quality appraisal. Country/region denotes the geographic origin of the study population; for systematic, scoping and narrative reviews that synthesise international evidence, it is reported as “multiple countries.” Setting denotes the care context (for example community dwelling, long-term care or home care) and is distinct from country/region.

Ref.	Author (Year), Country	Design	Setting	Focus	Key Finding Relevant to this Review	Main Limitation
[4]	Chan (2021), multiple countries	Systematic review	Mixed settings	Burden	High caries prevalence in older adults worldwide	Heterogeneous data
[5]	Huang (2025), multiple countries	Review (epidemiology)	General older population	Burden	Prevalence and risk factors of oral disease in older age	Non-systematic
[6]	Kocher (2021), Germany	National survey analysis (DMS)	Community dwelling (aged 65 to 74)	Burden	~half of 65–74 y affected by periodontitis; documents trends	Country-specific
[12]	Sahab (2025), multiple countries	Systematic review of longitudinal studies	Community and mixed	Outcomes	Oral health predicts healthy-ageing outcomes over time	Few longitudinal studies
[13]	Yu (2024), multiple countries	Scoping review	Mixed settings	OHL	Higher OHL links to better behaviours and outcomes; low OHL to misconceptions	Varied OHL instruments
[14]	Tenani (2020), Brazil	Cross-sectional	Community dwelling	OHL	Low OHL independently predicts dissatisfaction with oral health	Cross-sectional; no causality
[11]	Gopalakrishnan (2019), New Zealand	Cross-sectional survey	Residential aged care (rest homes); dependent	Barrier	Access barriers to oral care in residential aged care	Manager-reported
[15]	Beaven (2024), UK	Scoping review	Community and residential	Barrier	Maps barriers and facilitators to dental access	UK-specific
[16]	Ghanbari-Jahromi (2023), multiple countries	Scoping review	Mixed settings	Barrier	Utilisation driven more by systemic than individual factors	No formal appraisal
[17]	Zare (2024), multiple countries	Systematic review of reviews	Mixed settings (special needs)	Barrier	Lack of insurance among strongest utilisation barriers	Review of reviews
[18]	Bakker (2024), multiple countries	Scoping review	Nursing homes	Institutional	Poor oral health and assessment gaps in nursing homes	No meta-analysis
[19]	Ruiz-Roca (2021), multiple countries	Systematic review	Institutionalised/long-stay	Institutional	Poor oral status; worse than community-dwelling peers	Heterogeneous
[20]	Pombo-Lopes (2025), multiple countries	Systematic review	Nursing homes (residents and staff)	Institutional	Staff training and knowledge gaps act as barriers	Perception-based
[21]	Koistinen (2021), Sweden	Qualitative study	Short-term care facilities	Institutional	Lived experience of assisted daily oral care	Limited transferability
[22]	Julkunen (2024), Finland	Cross-sectional (clinical)	Long-term care (dentate and edentate)	Institutional	Oral frailty common; linked to oral disease burden	Single setting
[23]	Weening-Verbree (2024), multiple countries	Systematic review & meta-analysis	Long-term care facilities	Intervention	Multifaceted implementation strategies most effective	Implementation heterogeneity
[24]	Desai (2023), multiple countries	Narrative review	Mixed settings	Interaction	ROHD triggered by acute events; buffering-capacity concept	Non-systematic
[25]	Jamieson (2024), Australia & USA	Comparative decomposition analysis	Community (by race/ethnicity)	Interaction	Inequities sustained by structural and socioeconomic factors	Two-country scope
[26]	Lim (2021), South Korea	Longitudinal cohort	Community dwelling	Outcome	Poor oral health predicts geriatric syndromes, institutionalisation, mortality	Self-reported oral health
[27]	Henni (2023), multiple countries	Scoping review	Home healthcare	Outcome	Maps OHRQoL among home-care recipients	Descriptive
[7]	Borg-Bartolo (2025), multiple countries	Systematic review & meta-analysis	Mixed settings	Outcome	OHRQoL associated with structural determinants of health	Observational inputs
[28]	Azami-Aghdash (2021), multiple countries	Systematic review & meta-analysis	Mixed settings	Outcome	Documents burden on oral health-related quality of life	Heterogeneous
[29]	Aquilanti (2020), multiple countries	Systematic review	Mixed settings	Intervention	Teledentistry can improve access to care	Emerging evidence
[30]	Beltrán (2025), Chile	Implementation study	Rural/remote	Intervention	Satellite teledentistry improves access	Early stage
[31]	Mirwald & Foltin (2025), not reported	Cross-sectional comparative	Community, partially dependent and institutionalised	Interaction	Periodontitis far more prevalent in institutionalised group	Single study

**Table 2:** Conceptual stratification of older adults by functional status, with corresponding accumulation of oral health risk arising from literacy deficits and structural barriers.

Group	Description	Risk
A	Independent	Low
B	Assisted	Moderate
C	Institutionalised	High

## Data Availability

No new data were created or analysed in this study. Data sharing is not applicable for this article.

## Abbreviations

The following abbreviations are used in this manuscript:

DMS V	Fifth German Oral Health Study
LTC	Long-Term Care
NGO	Non-Governmental Organisation
OHL	Oral Health Literacy
OHRQoL	Oral Health-Related Quality of Life
PRISMA	Preferred Reporting Items for systematic Reviews and Meta-Analyses
ROHD	Rapid Oral Health Deterioration
SANRA	Scale for the Assessment of Narrative Review Articles
